# Supplementary material for: New Analytical Approach to Quinolizidine Alkaloids and Their Assumed Biosynthesis Pathways in Lupin Seeds
Source: Toxins (Basel). 2024 Mar 21;16(3):163. doi: 10.3390/toxins16030163 (PMC10974633; doi:10.3390/toxins16030163)
Supplement: Supplementary file 1 [file toxins-16-00163-s001.zip › toxins-2887398-supplementary.pdf]

Supplementary Material

Table S1a. Analytical QA standards.

Quinolizidine alkaloid	CAS no.	Supplier[a]	Article code	Purity (%)
(-)-Albine hydrochloride	53915-26-7	PhytoLab	86291	>98%
(-)-Anagyrine hydrochloride	74195-83-8	PhytoLab	83211	99.4%
(-)-Angustifoline	550-43-6	PhytoPlan	Art. 6320.95	99.2%
(-)-Cytisine	485-35-8	PhytoLab	80401	99%
(+)-Epilupinine chloride	486-71-5	PhytoPlan	n/a	>90%
Gramine	87-52-5	PhytoLab	80465	98%
(+)-13α-Hydroxylupanine	15358-48-2	PhytoPlan	Art. 6321.95	97.6%
(+)-α-Isolupanine	486-87-3	LGC	TRC-1820900	98%
(+)-Lupanine hydrochloride	550-90-3	PhytoPlan	Art. 6351.95	96.2%
(-)-Lupinine	486-70-4	PhytoLab	86294	97.1%
(-)-N-Methylcytisine	486-86-2	PhytoLab	83219	99.8%
(-)-Multiflorine	529-80-6	PhytoPlan	Art. 6324.95	99.6%
(-)-Sparteine	90-39-1	Sigma Aldrich	76466	98%
(-)-Thermopsine	486-90-8	MCE	HY-N5009	99.4%
13α-*trans*-Cinnamoyloxylupanine	5835-04-1	Biosynth	FC65854	>98%

a: Supplier details:
PhytoLab, Vestenbergsgreuth, Germany
PhytoPlan, Heidelberg, Germany
LGC Standards, Wesel, Germany
Sigma Aldrich, Zwijndrecht, The Netherlands
MCE: MedChemExpress LLC, Monmouth Junction, NJ, USA
Biosynth, Compton, United Kingdom

Table S1b. QAs tentatively identified in the samples.

Quinolizidine alkaloid	CAS no.
Isoangustifoline	82189-28-4
12,13-seco-12,13-didehydromultiflorine	6822-63-5
Tetrahydrorhombifoline	3382-84-1
5,6-Didehydromultiflorine	66216-62-4
13α-Hydroxymultiflorine	71657-64-2
3β-Hydroxylupanine	129443-39-6
3β,13α-Dihydroxylupanine	101512-24-7
13α-Tigloyloxylupanine	57943-34-7
13α-Angeloyloxylupanine	72822-06-1
13α-Tigloxymultiflorine	136396-56-0
13α-Angeloyloxymultiflorine	n.a.
13α-*cis*-Cinnamoyloxylupanine	86707-49-5

Table S2. Validation results: recovery and repeatability (n=6) for soybeans spiked at three concentration levels.

Level spiked (mg/kg)	1 mg/kg		5 mg/kg		25 mg/kg	
**Quinolizidine alkaloid**	**Recovery (%, n=6)**	**Relative standard deviation (%, n=6)**	**Recovery (%, n=6)**	**Relative standard deviation (%, n=6)**	**Recovery (%, n=6)**	**Relative standard deviation (%, n=6)**
Gramine	79.0	9.1	82.7	5.9	88.9	3.5
Epilupinine	91.8	11.6	96.0	10.9	91.9	7.6
Lupinine	90.9	8.1	99.2	10.7	95.9	3.2
Cytisine	99.7	13.7	99.2	7.6	98.3	5.5
Methylcytisine	87.3	8.0	92.1	4.6	87.8	2.1
Albine	88.5	8.1	95.5	7.6	96.5	3.6
Angustifoline	89.0	6.7	94.9	6.9	91.8	4.6
Sparteine	82.0	7.5	87.6	4.3	87.9	4.6
Anagyrine	92.8	6.7	99.9	8.0	93.2	4.5
Thermopsine	88.7	6.1	96.4	6.6	93.2	3.9
Multiflorine	89.9	6.7	93.7	4.9	92.7	4.2
Lupanine	93.0	9.6	94.9	5.6	94.9	5.0
Isolupanine	98.9	5.9	104.9	6.2	100.5	3.7
13-Hydroxylupanine	93.3	7.1	99.9	6.8	99.1	4.7
13-*trans*-Cinnamoyloxylupanine	53.5	2.9	49.7	3.7	44.2	7.1

Table S3. Linearity of calibration lines in blank soybean extract, 0-200 ng/mL, 9-point calibration, combined lines before and after analysis of the samples.

Quinolizidine alkaloid	Slope (cts/ng-mL)	Intercept (cts)	Correlation coefficient
Gramine	9520	-12246	0.9993
Epilupinine	6352	11165	0.9988
Lupinine	35879	31710	0.9988
Cytisine	36128	99120	0.9968
Methylcytisine	22074	83800	0.9960
Albine	36195	23801	0.9996
Angustifoline	41150	27440	0.9987
Sparteine	45416	5477	0.9991
Anagyrine	55660	119139	0.9982
Thermopsine	48218	98798	0.9980
Multiflorine	66602	46408	0.9994
Lupanine	64045	23306	0.9991
Isolupanine	84784	61504	0.9992
13-Hydroxylupanine	59063	38693	0.9987
13-*trans*-Cinnamoyloxylupanine	63428	17095	0.9954

Table S4. Quinolizidine alkaloids were detected in the lupin species and control plants when analyzed using both LC-MS/MS and GC/MS. Amounts are given in mg/kg of seed dry weight.

Species	Population	Total QAs	Epilupinine	Lupinine	Sparteine	Multiflorine	11,12-seco-12,13-dehydromultiflorine*	13α-Hydroxymultiflorine*	13α-Tigloyloxymultiflorine*	13α-Angeloyloxymultiflorine*	5,6-Didehydromultiflorine*	Albine	Angustifoline	Isoangustifoline*	Lupanine	Isolupanine	3β-Hydroxylupanine*	13α-Hydroxylupanine	3β,13α-Dihydroxylupanine*	13α-Tigloyloxylupanine*	13α-cis-cinnamoyloxylupanine*	Gramine	Tetrahydrorhombifoline*
L. pilosus	PIL-01	11471	2250	11	26	8328	239	349	200	6	44	6	0	0	7	0	0	0	0	0	0	0	0
L. pilosus	PIL-02	9739	2866	11	201	6005	338	178	79	2	35	8	0	0	8	0	0	0	0	0	0	0	0
L. pilosus	PIL-03	10725	3184	11	393	6647	226	166	46	1	34	6	0	0	5	0	0	0	0	0	0	0	0
L. pilosus	PIL-04	9172	2039	9	123	6389	245	219	94	2	31	7	0	0	8	0	0	0	0	0	0	0	0
L. pilosus	PIL-05	11618	3304	16	64	7257	262	421	213	8	51	8	0	0	7	0	0	0	0	0	0	0	0
L. pilosus	PIL-10	10584	2874	9	9	7016	145	326	141	4	45	3	0	0	6	0	0	0	0	0	0	0	0
L. pilosus	PIL-11	10421	3891	14	466	5463	297	171	59	1	37	9	0	0	7	0	0	0	0	0	0	0	0
L. pilosus	PIL-13	10714	2348	19	22	7714	45	323	175	6	49	1	0	0	7	0	0	0	0	0	0	0	0
L. pilosus	PIL-14	11166	2507	11	13	8017	173	263	135	3	28	3	0	0	6	0	0	0	0	0	0	0	0
L. pilosus	PIL-18	9521	2518	8	459	6146	178	116	53	3	30	4	0	0	3	0	0	0	0	0	0	0	0
L. pilosus	PIL-19	10222	2630	8	158	6973	259	118	35	2	24	5	0	0	5	0	0	0	0	0	0	0	0
L. pilosus	PIL-20	9802	2533	7	10	6680	159	262	109	5	25	3	0	0	4	0	0	0	0	0	0	0	0
L. pilosus	PIL-21	9122	3182	7	108	5038	466	211	63	3	23	12	0	0	4	0	0	1	0	0	0	0	0
L. pilosus	PIL-23	9212	2914	9	196	5578	247	171	47	2	28	8	0	0	6	0	0	1	0	0	0	0	0
L. pilosus	PIL-25	10380	3152	11	264	6525	176	149	42	3	42	7	0	0	6	0	0	1	0	0	0	0	0

L. pilosus	PIL-26	12193	3422	9	285	8223	96	76	25	1	41	4	0	0	5	0	0	1	0	0	0	0	0
L. pilosus	PIL-30	9889	3277	9	187	5434	613	239	76	4	24	14	0	0	5	0	0	1	0	0	0	0	0
L. pilosus	PIL-33	8383	2741	11	241	4774	325	178	64	3	23	8	1	0	7	0	0	2	0	0	0	0	0
L. pilosus	PIL-34	10318	2558	9	113	7030	280	213	63	3	26	9	0	0	7	0	0	1	0	0	0	0	0
L. pilosus	PIL-36	11242	2150	8	130	8297	260	284	68	3	25	6	0	0	5	0	0	1	0	0	0	0	0
L. palaestinus	PA-02	6964	419	3	339	5845	162	115	42	0	29	4	0	0	3	0	0	1	0	0	0	0	0
L. palaestinus	PA-03	6656	490	4	247	5666	112	79	33	0	18	2	0	0	3	0	0	1	0	0	0	0	0
L. palaestinus	PA-04	3990	783	4	300	2776	38	53	17	0	15	1	0	0	2	0	0	1	0	0	0	0	0
L. palaestinus	PA-05	4858	320	2	206	4100	96	72	36	0	16	2	0	0	4	0	0	2	0	0	0	0	0
L. palaestinus	PA-07	6975	300	2	281	6117	100	99	40	0	26	3	0	0	4	0	0	1	0	0	0	0	0
L. palaestinus	PA-10	6803	261	3	281	6002	103	82	34	0	21	3	1	0	7	0	0	4	0	0	0	0	0
L. palaestinus	PA-11	6200	322	3	245	5392	99	79	31	0	22	2	0	0	3	0	0	1	0	0	0	0	0
L. palaestinus	PA-12	5973	479	2	192	5065	96	81	33	0	18	2	0	0	3	0	0	1	0	0	0	0	0
L. palaestinus	PA-15	5532	450	3	207	4686	60	81	23	0	16	1	0	0	3	0	0	1	0	0	0	0	0
L. palaestinus	PA-16	5203	1039	4	149	3803	92	62	27	0	13	3	1	0	7	0	0	3	0	0	0	0	0
L. palaestinus	PA-17	7788	1016	7	230	6181	112	92	38	0	23	12	4	0	47	0	1	18	0	1	0	0	0
L. albus-bitter	ALB-05	23876	0	0	16	936	711	1087	57	40	9	2352	640	20	13092	85	209	4282	0	300	0	0	40
L. albus-bitter	ALB-07	25310	0	0	20	713	575	1006	47	40	8	2338	645	21	14345	88	229	4857	1	332	0	0	45

L. albus-sweet	ALB-12	96	0	0	0	2	11	5	1	0	0	8	4	0	31	1	2	20	0	8	0	0	1
L. albus-sweet	ALB-sh	95	0	0	0	4	8	3	1	0	0	3	8	0	29	2	2	26	0	6	0	0	1
L. angusti-folius	ANG-sh	32840	12	0	5	522	127	77	1	0	3	21	7243	89	6384	68	48	1805<br>2	1	26	153	0	8
L. luteus	LUT-03	17106	468	13911	2	59	3	1	0	0	0	0	11	0	5	0	0	38	0	0	0	2606	0
L. mutabi-lis	MUT-01	24536	5	23	6794	49	2	1	0	0	0	0	20	1	11016	95	9	3201	1432	341	741	15	791
*G. max***	MAX-01	10	0	0	2	1	0	0	0	0	0	0	0	0	4	0	0	1	1	0	1	0	0
*C. ari-etinum***	ARI-01	7	0	0	1	3	0	0	0	0	0	0	0	0	2	0	0	1	0	0	0	0	0

* Semi-quantitative results. ** The low levels of QAs detected in *G. max* and *C. arietinum* are likely due to cross-contamination caused by the *L. mutabilis* sample during sample preparation/analysis.

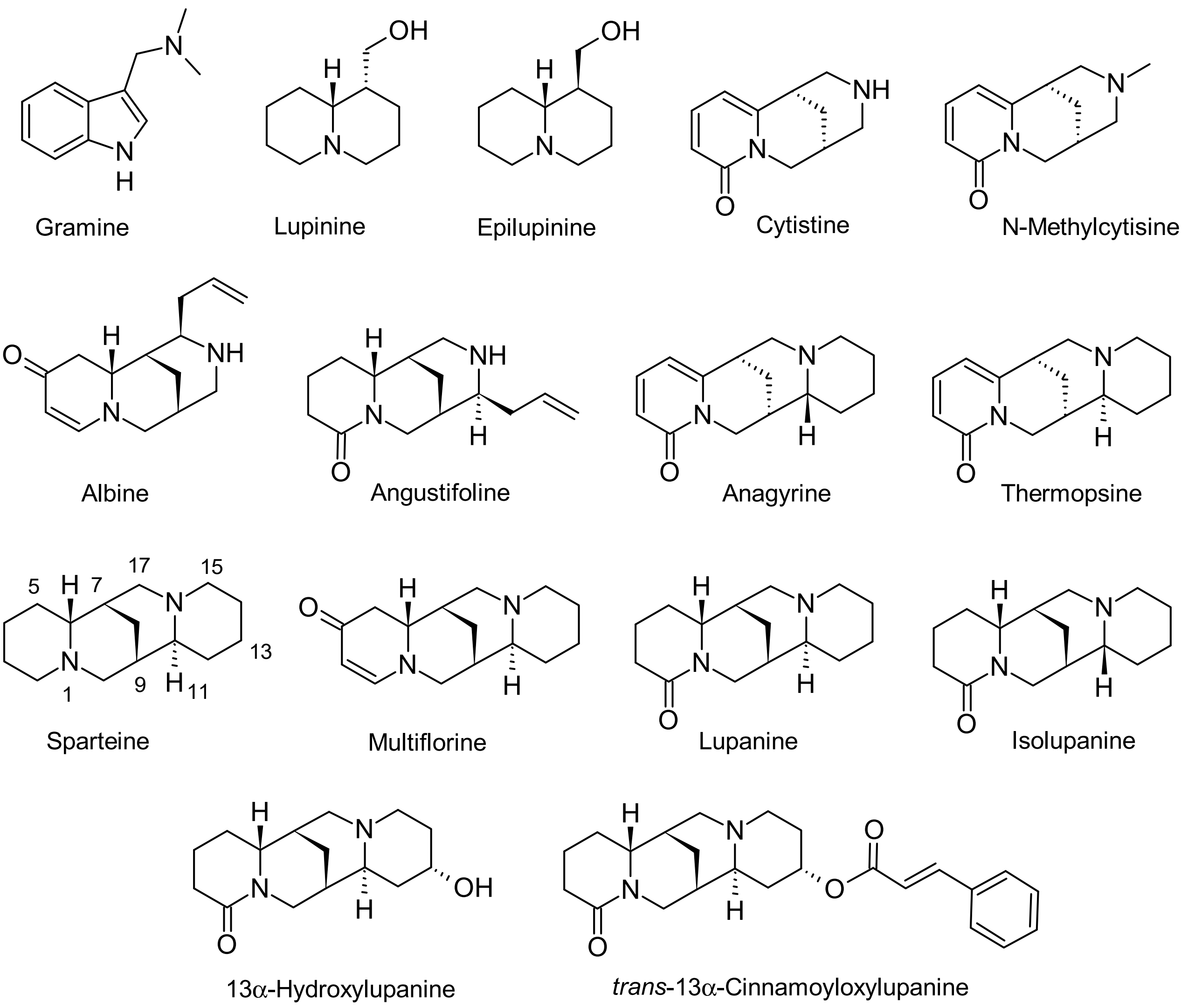

Supplementary Figure S1A: Quinolizidine alkaloid reference standards.

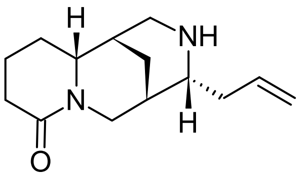

Isoangustifoline

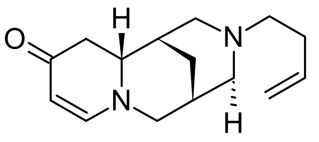

11,12-*seco*-12,13-Didehydromultiflorine

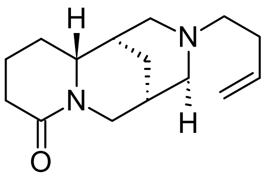

Tetrahydrorhombifoline

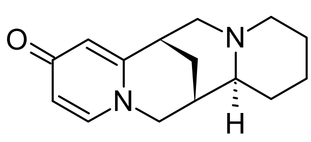

5,6-Didehydromultiflorine

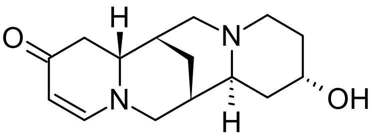

13α-Hydroxymultiflorine

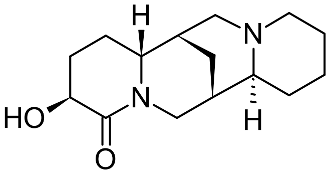

3β-Hydroxylupanine

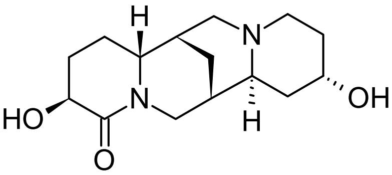

3β,13α-Dihydroxylupanine

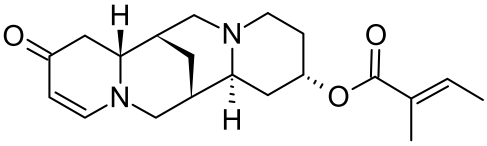

13α-Tigloyloxymultiflorine

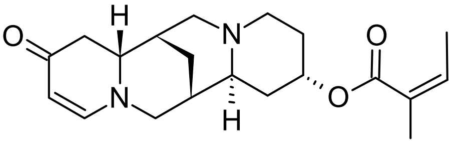

13α-Angeloyloxymultiflorine

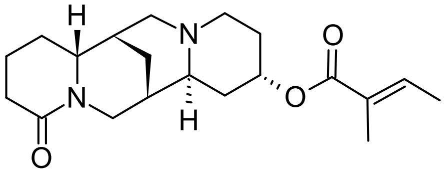

13α-Tigloyloxylupanine

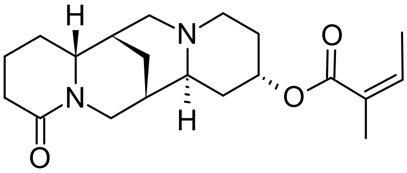

13α-Angeloyloxylupanine

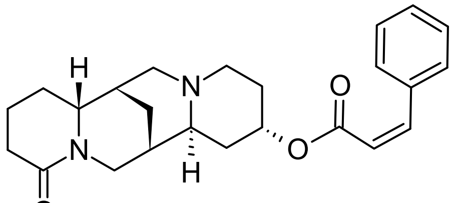

cis-13α-Cinnamoyloxylupanine

Supplementary Figure S1B: Additional quinolizidine alkaloids included in the method.

Supplementary Figures S2A-G. Representative LC-MS/MS MRM chromatograms of the six main lupin species were analyzed.

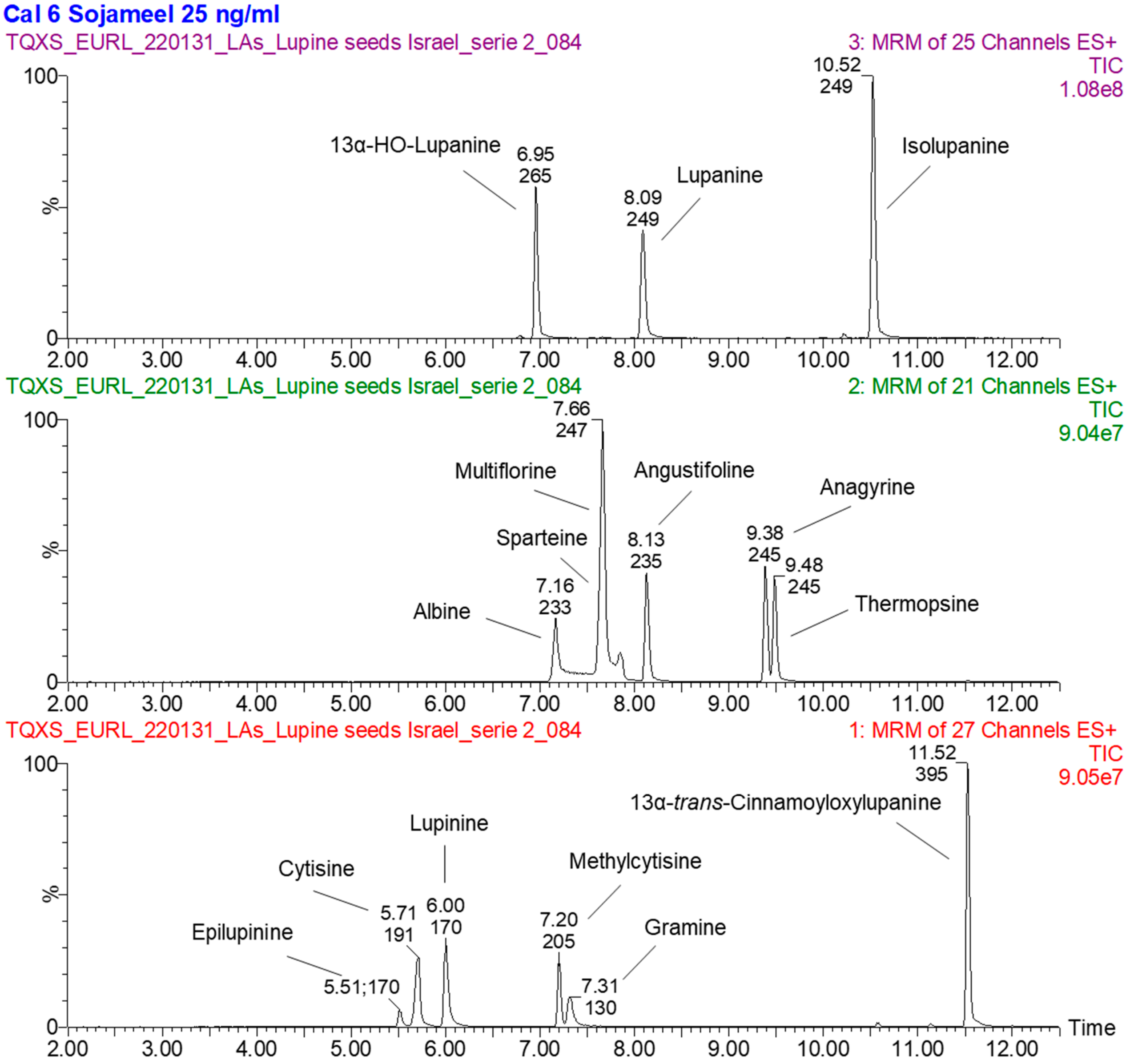

Supplementary Figure S2A. A mixture of QA reference standards (25 ng/mL in blank soy extract).

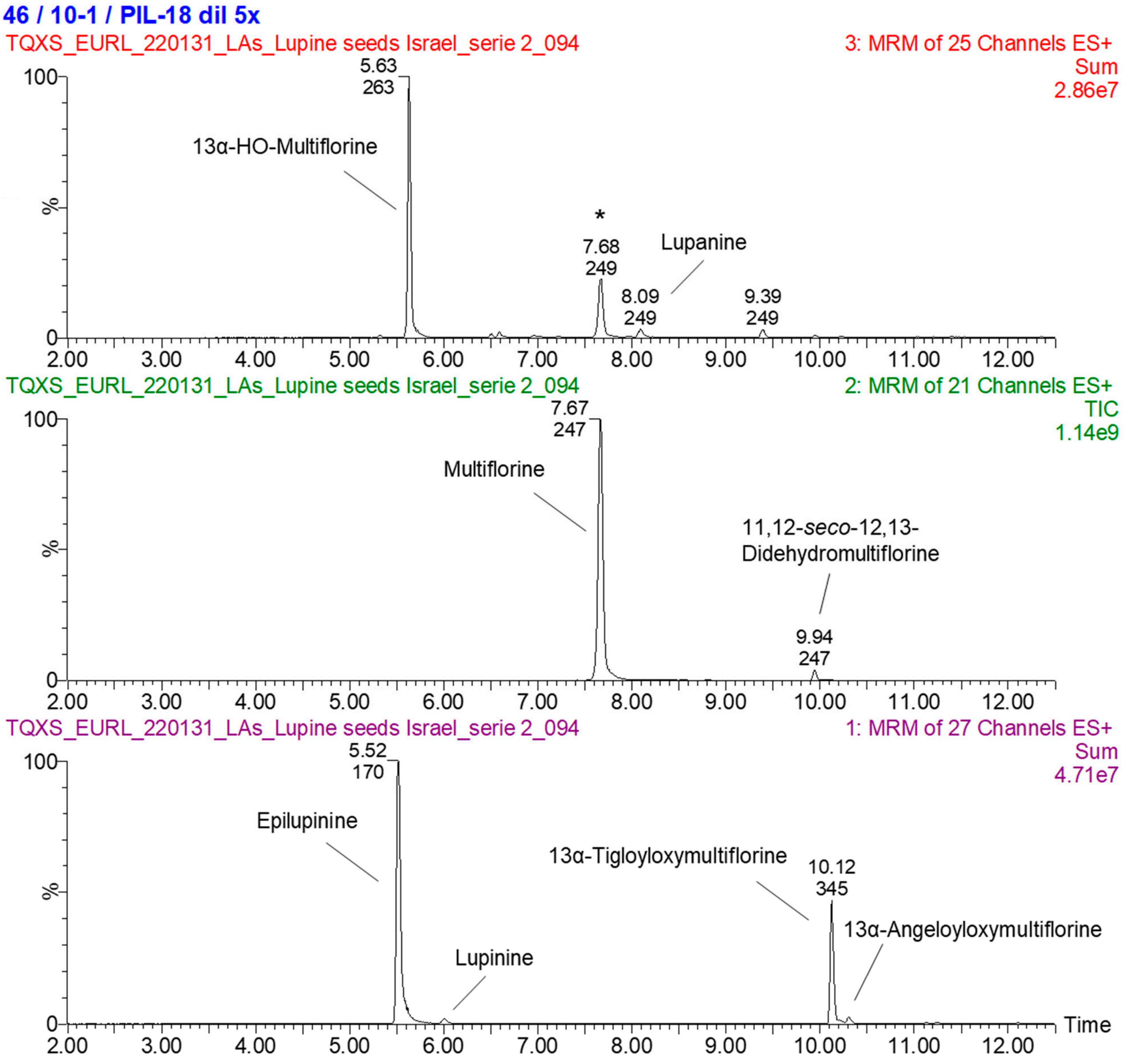

Supplementary Figure S2B: *Lupinus pilosus* (PIL-18).

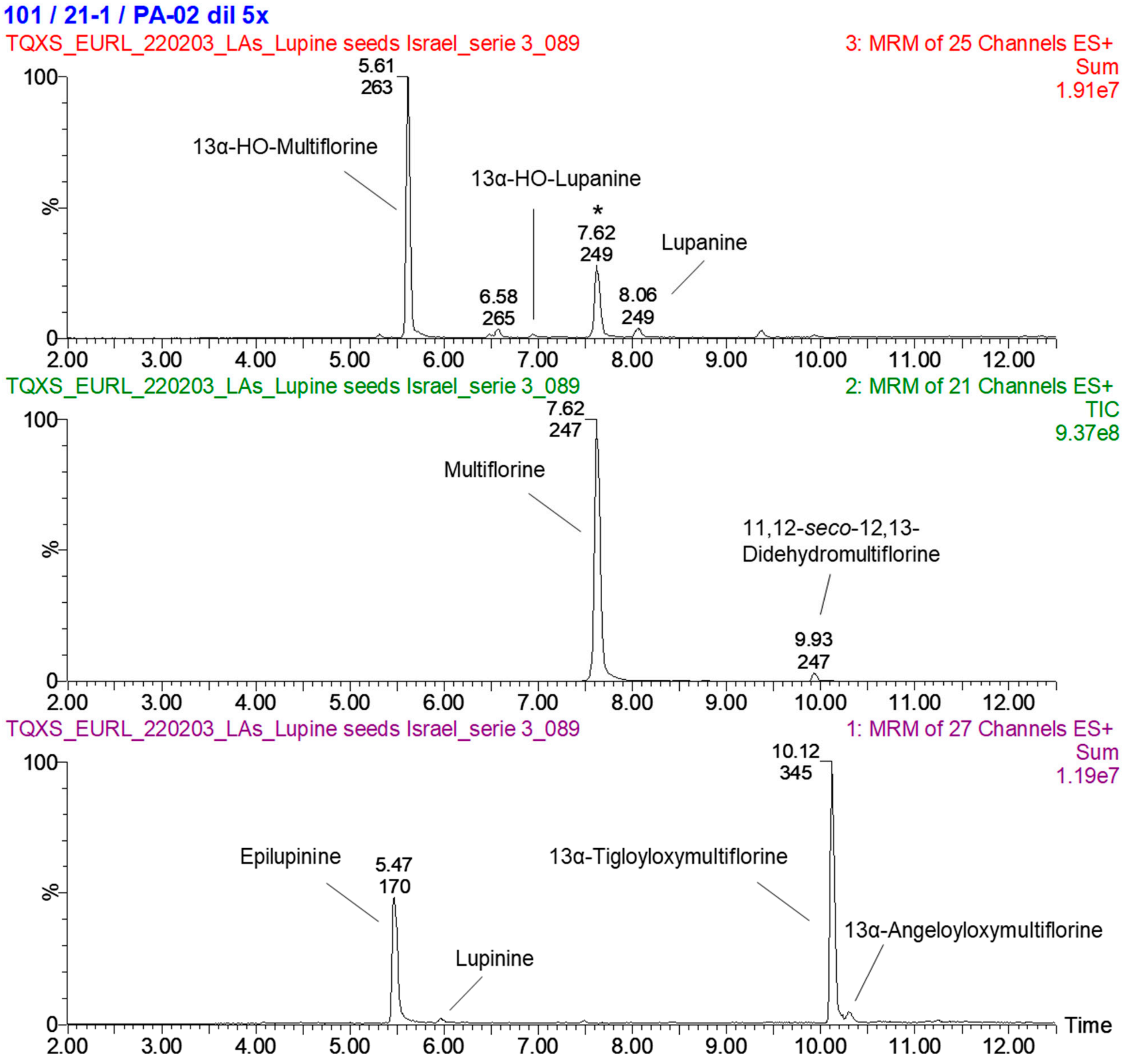

Supplementary Figure S2C: *Lupinus palaestinus* (PA-02).

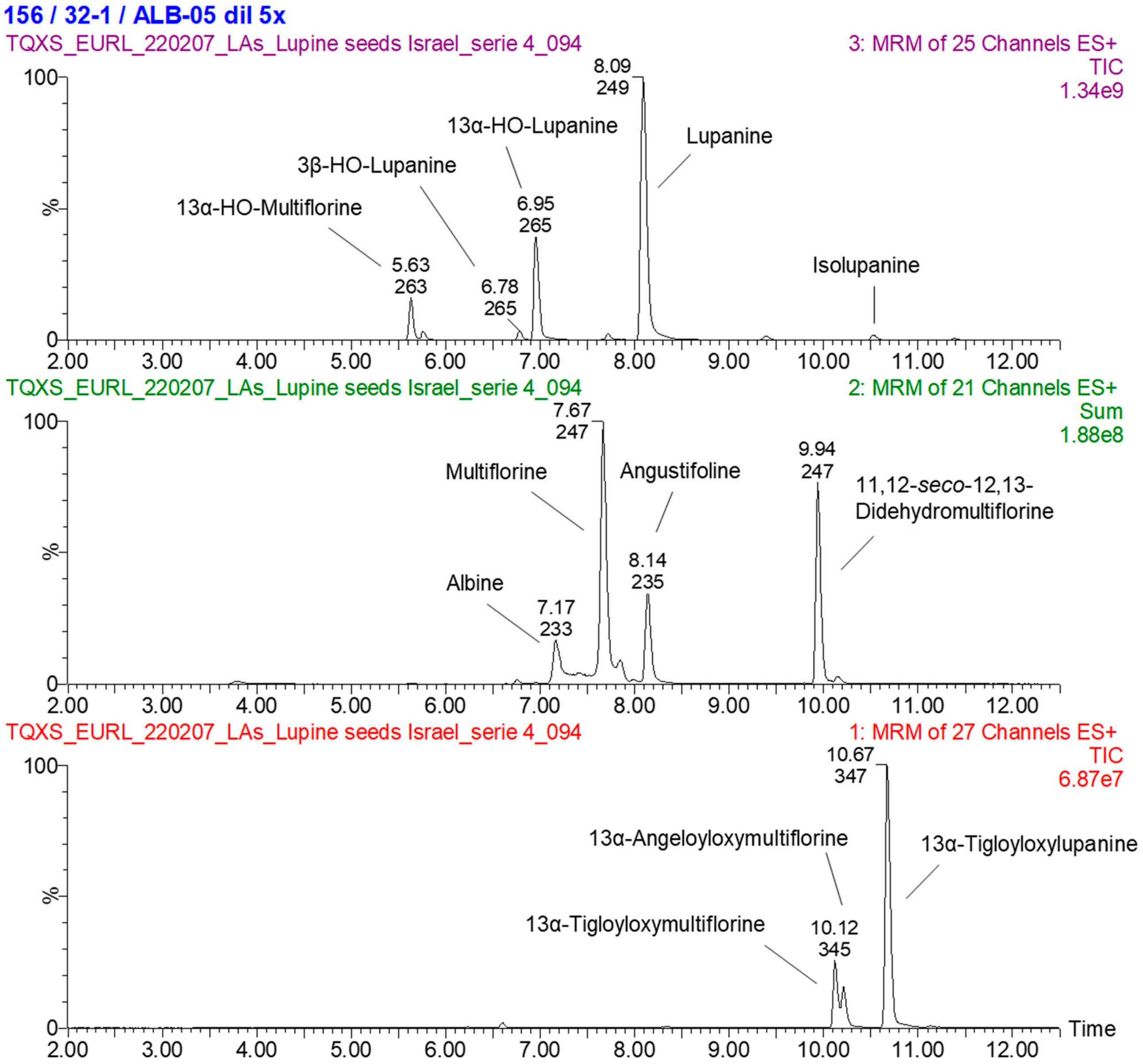

Supplementary Figure S2D: *Lupinus albus* (ALB-05).

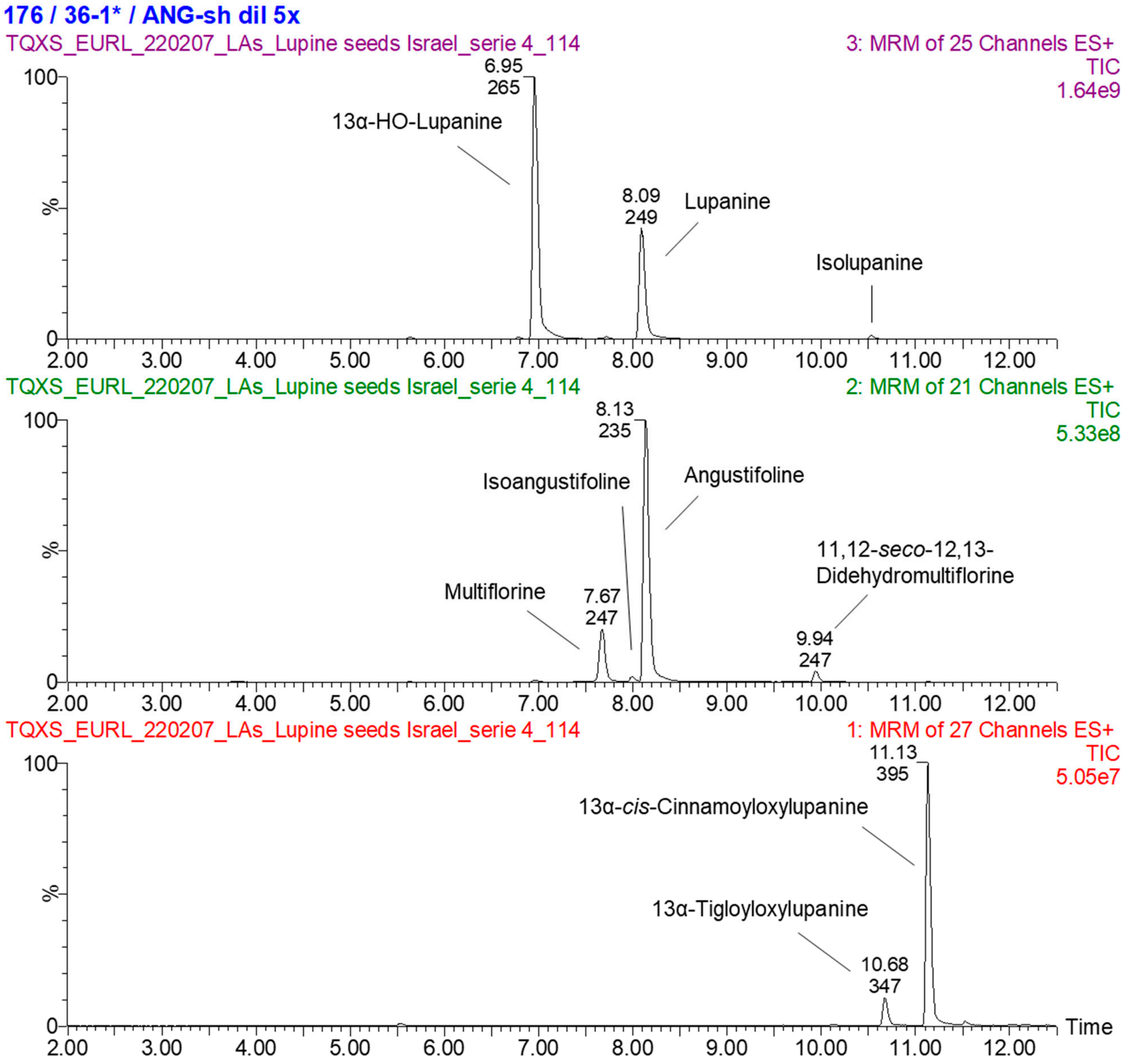

Supplementary Figure S2E: *Lupinus angustifolius* (ANG-sh).

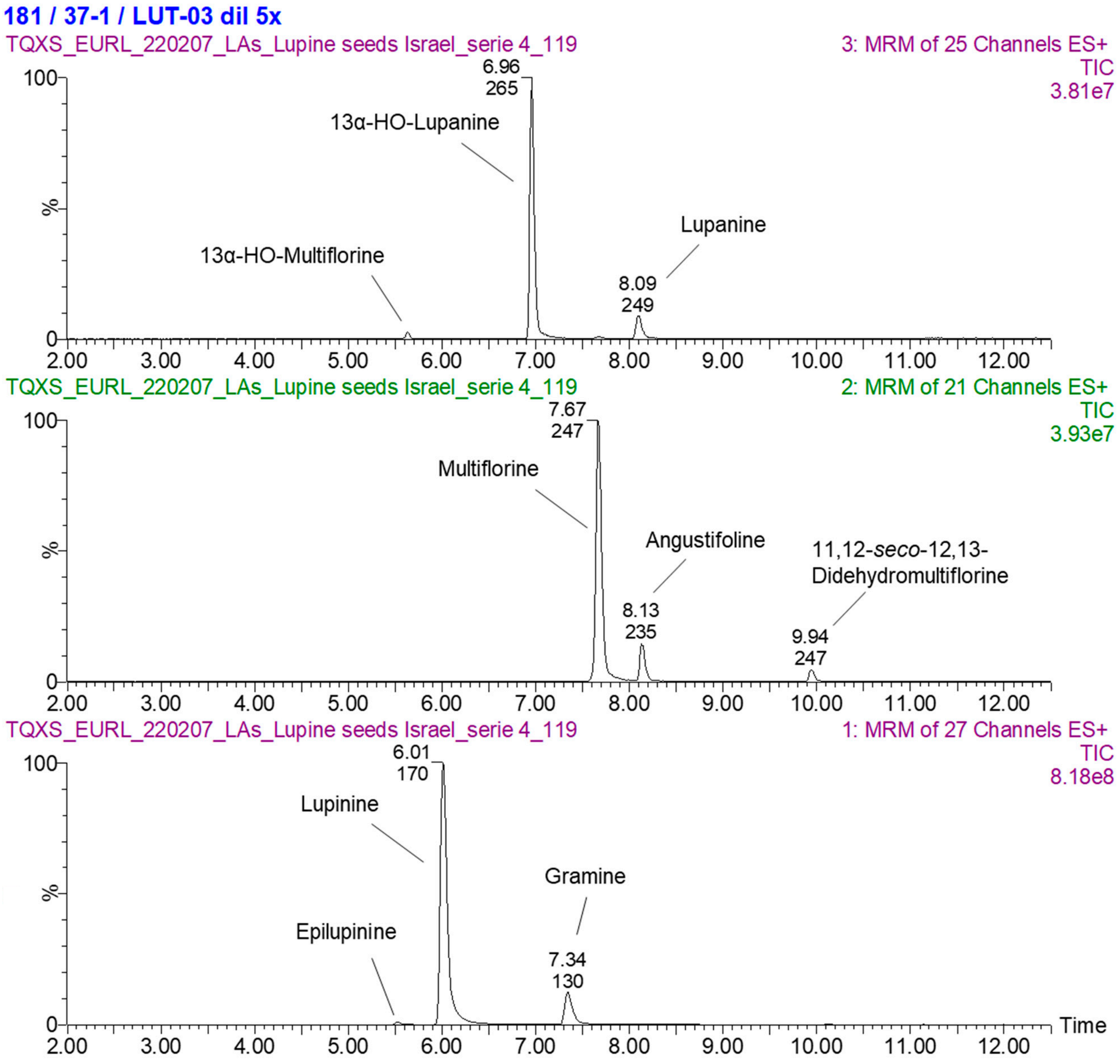

Supplementary Figure S2F: *Lupinus luteus* (LUT-03).

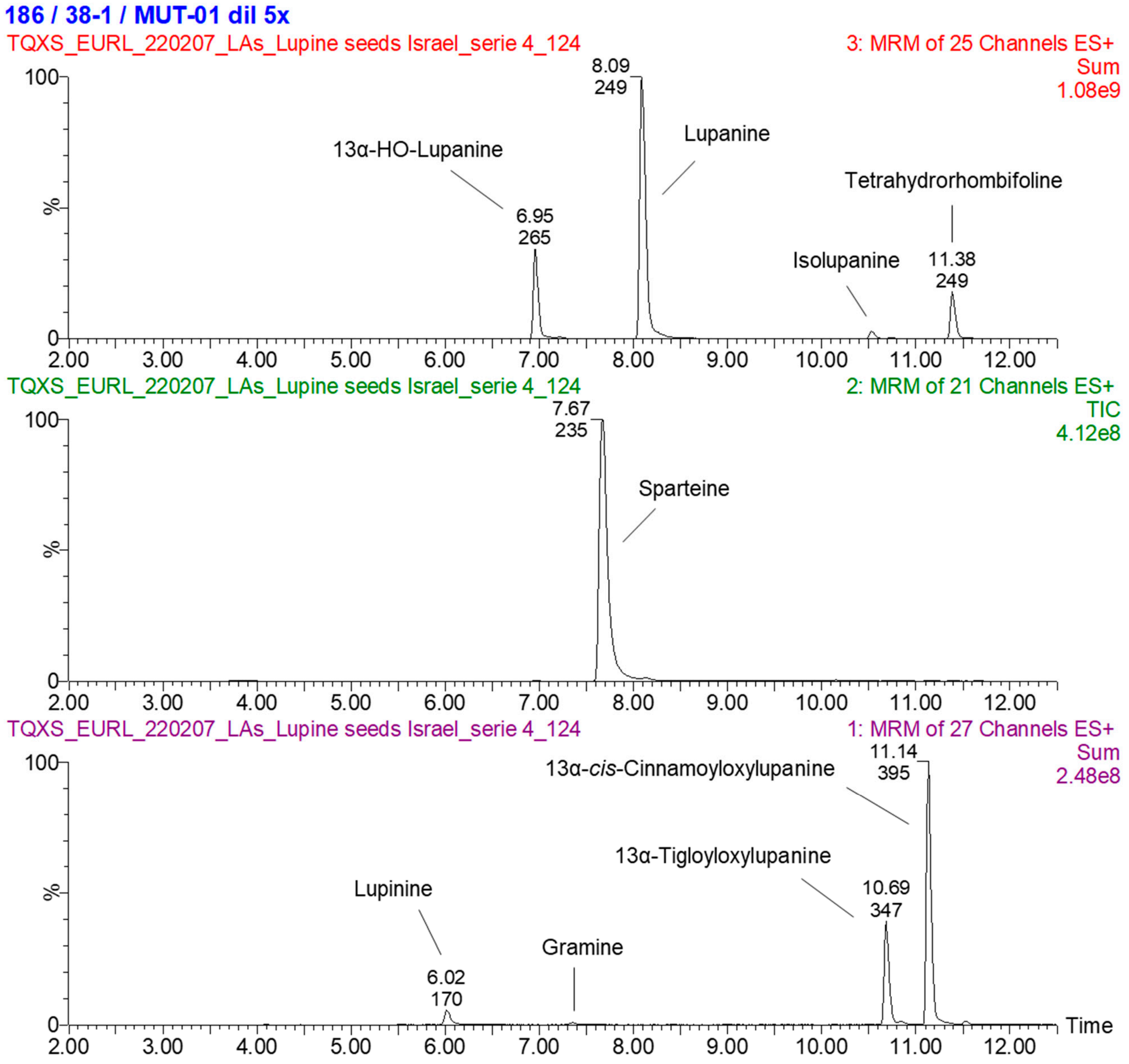

Supplementary Figure S2G: *Lupinus mutabilis* (MUT-01).
